# The Annual Meeting of the Lake Erie Dental Association

**Published:** 1872-04

**Authors:** 


### The Annual Meeting of the Lake Erie Dental Association

Will be held at the Universalist Church, Conneautville, Pa., commencing Tuesday, May 7th, at 10 A. M. Session to continue three days. Members and visitors can find good accommodations at the Power and Holman Houses, for $1.50 per day. Trains from the North arrive at Conneautville at 12 noon, and 6 P. M. Trains from the South arrive at 1 and 10 P. M. J. G. TEMPLETON, Pres't, New Castle, Pa.

C. B. Ansart, Sec'y, Oil City, Pa.

### SUBJECTS OF ESSAYS FOR DISCUSSION.

1st. Mchanical Dentistry, S. W. Tenney, Corry, Pa., Essayist; 2d. Operative Dentistry, especially filling of teeth, C. D. Elliott, Franklin, Pa., Essayist; 3d. Treatment of Children's Teeth, W. W. Martin, Mercer, Pa., Essayist; 4th. Treatment of Diseased Gums, C. H. Griffin, Oil City, Pa., Essayist; 5th. Dental Pathology, W. T. Wallace, Kingsville, Ohio, Essayist; 6th. Treatment of Dental Pulps, D. C. Dunn, Meadville, Pa., Essayist; 7th. Why should we Extract Teeth; S. B. Humes, Edinboro, Pa., Essayist; 8th. Dental Caries, W. E. Magill, Erie, Pa.. Essayist.

Dr. A. B. Robbins, of Meadville, will deliver a public address, Wednesday evening, May 8th, at the Universalist Church. Subject: Dental Education.

